# Investigation of primary health care service delivery models used in allied health practice in rural and remote areas of Australia: a systematic review

**DOI:** 10.1186/s12913-025-12717-6

**Published:** 2025-04-21

**Authors:** Alison Brown, Alexandra Cant, Rebecca Wolfgang, Robyn Ramsden, Susan Heaney, Leanne J. Brown

**Affiliations:** 1 University of Newcastle, Callaghan, Australia; 2 Hunter New England Population Health, Wallsend, Australia; 3 Physio Inq, Sydney, Australia; 4 Rebecca Wolfgang Consulting, Newcastle, Australia; 5 Rural Doctors Network, St Leonards, Australia; 6 Deakin University, Burwood, Australia; 7 Hunter Medical Research Institute, New Lambton, Australia

**Keywords:** Access, Allied health professions, Health promotion, Models of care, Remote, Rural, Systematic review, Quality of life, Primary health care

## Abstract

**Introduction:**

In Australia, access to primary health care (PHC) services is limited in comparison to major cities. Allied health professionals play a pivotal role in providing necessary PHC in rural and remote areas. However, there is limited evidence about the most effective allied health specific PHC models of care that can be utilised in these settings. The aim of this review was to describe the PHC models used by allied health professionals in rural and remote areas of Australia and report on their impact and effectiveness in improving care.

**Methods:**

A search of five databases (MEDLINE, Embase, CINAHL, PsychINFO and Informit Health) was undertaken. Articles were included that related to a refined list of allied health professions that specifically work in PHC settings, these included: dietetics; occupational therapy; physiotherapy; psychology; speech pathology; social work; podiatry; exercise physiology; pharmacy; optometry; and audiology. Articles with a focus on a PHC model of service delivery in a rural or remote area were included. The effectiveness and impact of these models was examined. The Mixed Methods Appraisal Tool was used to assess the quality of the included articles.

**Results:**

A total of 57 articles met the inclusion criteria, from an initial 1864 unique citations sourced from searches. Of the 57 articles, 22 studies were in the Australian context and were included in this paper. The outcome measures typically included improving access to services, however minimal impact or effectiveness data was reported. Studies were categorised into an existing typology of PHC models: integrated services (*n* = 9); outreach services (*n* = 3); virtual outreach services (*n* = 4); discrete services (*n* = 4); with an additional model being health promotion (*n* = 5).

**Conclusion:**

A range of PHC models were used by allied health disciplines in rural and remote areas of Australia. These models focused on improving access to allied health services in primary care settings to address health inequities faced. Given the limited reporting of the impact of these services, it is recommended that rigorous evaluations of existing allied health models are undertaken. There is a gap in the literature regarding the models of service delivery being used by allied health professionals in non-metropolitan areas.

**Supplementary Information:**

The online version contains supplementary material available at 10.1186/s12913-025-12717-6.

## Background

Access to primary health care (PHC) is a fundamental human right and is considered essential for universal health coverage [1]. PHC relates to patient treatment that is delivered outside of the hospital setting and focuses on disease prevention, early intervention and management of existing conditions [2]. It is a vital component in delivering essential healthcare, particularly as the burden of non-communicable diseases increases, globally [3]. PHC has the ability to decrease hospital admissions and prevent avoidable readmissions [3]. Despite the strong evidence in support for PHC to improve health and wellbeing across all life stages, there continues to be significant challenges to providing PHC in rural and remote settings, globally and in Australia.

Approximately 28% of the Australian population live in rural and remote areas [4]. Those living in rural and remote areas often have poorer health outcomes in comparison to people living in metropolitan areas [4]. In Australia, the total burden of disease and injury has been reported to increase with an increase in remoteness. This includes the burden of disease for chronic diseases such as cardiovascular disease, diabetes, chronic kidney disease and respiratory conditions [4]. Inadequate access to PHC providers and health services in these areas is considered to be a leading cause of the health disparity for people living in rural and remote locations [4]. As such, there is a significant need to improve PHC provision in rural and remote Australia.

Models of care used in rural and remote communities may vary from those used in metropolitan areas to account for the limited access to PHC in these settings [5]. A model of care can be described as the way healthcare services are delivered [6]. The key elements in an effective model of care include: accessible, patient-centred, continuity of care, innovative, fit-for-purpose, efficient service delivery and effectively use available resources [5, 7, 8]. Factors that contribute to limited access to PHC in rural and remote areas include geographical isolation, reduced healthcare infrastructure and workforce shortages, which require tailored approaches to ensure equitable access to care [9, 10]. Types of rural PHC models include private allied health services, discrete services (including walk-in/walk-out), integrated services (such as shared care), comprehensive primary health care services (such as Aboriginal controlled community health services), virtual or telehealth services and face to face outreach services (including fly-in, fly-out) [11, 12]. In addition to adapting service models, improved access to PHC services in rural and remote settings can depend on the willingness of clinicians to use an extended scope of practice [8]. An extended scope of practice is where a health professional develops a broader skill set and therefore offers a wider range of services beyond their recognised scope of practice [13]. In rural areas, where a full range of services may not be available, extended scope of practice may be utilised by allied health professionals to increase the quality and breadth of services provided [14].

Allied health professionals, who provide essential services for preventative, early intervention and chronic disease management, are an important component of the PHC workforce [15]. At present, there is poor distribution of allied health professionals across Australia, with over 70% of all allied health professionals living and working in metropolitan locations, with the number of allied health practitioners decreasing with increasing remoteness [15]. This is often cited to be due to low retention and recruitment of allied health professionals to rural and remote settings [8, 16–18]. More recently, there has been national interest in improving the current allied health service distribution in these settings. Evidence of this can be seen in the allied health rural generalist program, where new graduates are supported to undertake generalist roles and undertake postgraduate qualifications in rural practice [16].

Whilst there is renewed focus on improving allied health services in rural and remote areas [16], there is a paucity of evidence around the most effective allied health specific PHC models of care that can be utilised in these settings. A scoping review explored aspects of service models used by allied health professionals to improve service distribution and reported that telehealth services for home-based cardiac rehabilitation was as effective as a face-to-face service [14]. Similarly a systematic review and meta-analysis reported telehealth interventions delivered by allied health professionals and nurses (with the majority being nurses and psychology professionals) were as effective as face to face interventions [18]. However, both reviews focussed on telehealth as a specific model of service delivery and did not consider other models of PHC [14]. A further two reviews explored PHC with a focus on general practice and were not specific to allied health [8, 11]. There have been no systematic reviews that focus specifically on allied health PHC models of service in regional, rural and remote areas. Given the gap in the literature, the aim of this systematic review was to investigate primary health care models of service used by allied health professionals in rural and remote areas, with a focus on the Australian context. The effectiveness and impact of these models was examined in terms of service access and availability, as well as quality of care and health outcomes. Specifically, the research questions were:

i) What primary health care models are used by allied health professionals in rural and remote areas of Australia?
ii) What was the impact of these primary health care models on service access and availability?
iii) What was the effect of these primary health care models on quality of care and/or health outcomes?

## Methods

A systematic review protocol was registered with PROPSPERO (CRD42021251696; 11/06/2021). The methodology and reporting for this systematic review followed the Preferred Reporting Items for Systematic Reviews and Meta-Analyses (PRISMA) checklist and statement [19].

### Eligibility criteria

Eligible articles focussed on PHC models that delivered services outside the acute hospital setting. Allied health disciplines were chosen based on the Services for Australian Rural and Remote Allied Health list of allied health professions [20] and this list was further refined to only include allied health professions that are directly involved in primary health care delivery. Therefore, articles were required to include services of one or more of the following allied health disciplines: dietetics; occupational therapy; physiotherapy; psychology; speech pathology; social work; podiatry; exercise physiology; pharmacy; optometry; and audiology. Geographic location was restricted to non-metropolitan areas, encompassing locations fitting the description of regional, rural or remote areas either by author description or according to the location of the service using the Modified Monash Model classification [21] as a guide. Studies were included if they provided comparisons with models used in metropolitan areas, if the rural or remote model data was discernible from any metropolitan model data. Studies reported in English and published from 2000 onwards were included, to ensure practice model currency and relevance. Only articles reporting primary research were included, inclusive of any type of research methodology and study design.

### Search strategy

A search strategy was developed in consultation with a university Research Liaison Librarian. Literature searching was conducted in five peer-reviewed databases: MEDLINE; Embase; CINAHL; PsychINFO; and Informit Health. The full search strategy of MEDLINE database is shown in Table 1, this search strategy was adapted for use with other databases. An initial search was conducted on 14 March 2021, with repeated searches on 24 May 2021, 17 November 2022 and 30 August 2024. ‘Grey’ literature was included from government websites, university organisations and works known to the research team. Hand searching was also completed by reviewing reference lists of included articles.

**Table 1:** Search strategy for this review in MEDLINE

#	Searches	Results
1	primary health care/or primary health.mp.	99,258
2	health promotion/or promotion.mp.	158,646
3	primary prevention/or prevention.mp.	1,722,610
4	1 or 2 or 3	1,916,133
5	rural.mp. or rural health/or rural health services/	176,173
6	models.mp.	2,568,983
7	4 and 5 and 6	3783
8	allied health personnel/or allied health.mp.	20,765
9	dietitian.mp. or nutritionists/	4229
10	dietician.mp. or nutritionists/	2314
11	occupational therapy/or occupational ther*.mp.	20,904
12	social work/or social work*.mp.	28,397
13	physio*.mp.	5,545,446
14	physical ther*.mp. or physical therapists/	57,952
15	Psycholog*.mp. or Psychology/	1,545,157
16	Pharma*.mp.	4,011,147
17	speech path*.mp.	1464
18	Language Therapy/or Speech Therapy/or speech ther*.mp.	10,080
19	physiotherapy.mp.	21,311
20	Optometrists/or Optometry/or optometr*.mp.	8047
21	Audiology/or audiolo*.mp. or Audiologists/	12,093
22	Exercise/or Physiology/or exercise physiolo*.mp.	142,738
23	Exercise Therapy/or Exercise/or exercise ther*.mp.	157,639
24	Mental Health/or Community Mental Health Services/or Mental Health Services/or mental.mp.	578,394
25	8 or 9 or 10 or 11 or 12 or 13 or 14 or 15 or 16 or 17 or 18 or 19 or 20 or 21 or 22 or 23 or 24	10,063,202
26	access.mp.	352,455
27	Treatment Outcome/or Outcome Assessment, Health Care/or outcome.mp.	2,024,212
28	“Quality of Health Care”/or “Quality of Life”/or quality.mp.	1,289,730
29	26 or 27 or 28	3,369,422
30	7 and 25 and 29	397
31	limit 30 to (english language and yr="2000 -Current”)	370

Result numbers are in accordance with the original search from 2021

### Study selection

Database searches were uploaded into Endnote (X9.2, Clarivate, Philadelphia, PA, USA) and duplicate articles removed before results were transferred into Covidence (Veritas Health Innovation, Melbourne, Victoria, Australia). Covidence was used to manage references for the review screening [22]. Two researchers (AC, LB, TS, RW, AB or SH) independently reviewed the title and abstract of included articles according to agreed inclusion and exclusion criteria (Table 2). Remaining full text articles were also screened by two researchers and any conflicts resolved by discussion and, if agreement was unable to be reached, a third researcher reviewed the article.

**Table 2:** Inclusion and exclusion criteria for the literature included in this review

Criteria	Inclusion	Exclusion
Time period	2000 onwards	Prior to 2000
Language	English language	Not in English language
Geographical delimitation	Rural or remote	No relevance to rural or remote areas
Aspect of health care	Primary health care model	Secondary and tertiary services
Allied health professions	Refined list of allied health professions (dietitians, occupational therapy, physiotherapy, psychology, speech pathology, social work, podiatrists, exercise physiologist, psychology, pharmacy, optometry, audiology) Models that have allied health professionals and this can be clearly identified, including models with other health professionals e.g. nurses, Aboriginal Health Workers	Health professions not included in the list. Reviews focusing specifically on nursing or medical models
Types of Studies to be included	Qualitative and quantitative primary studies; mixed method studies; randomised control trials, quasi-experimental design, pilot studies and case studies	Systematic reviews, conference abstracts, thesis and scoping reviews

### Data collection process and data items

A data extraction form was developed in Excel and used to extract information including title, authors, publication date, timing of the study, primary aim/s of study, location of service, demographic characteristics of the study sample, allied health professions included, type of primary health care service (as depicted by Table 3), primary and secondary outcomes measures and implications/recommendations. Data extraction was conducted by one researcher and independently checked by a second reviewer, with any conflicts resolved through discussion and reference to the data extraction tool.

**Table 3:** Existing typology of rural and remote primary health care models [11]

Model Category	Health Service Model Examples
Discrete Services	Walk-in/Walk out
Integrated Services	Shared care Primary Health Care teams
Comprehensive Primary Health Care Services	Aboriginal Controlled Community Health Services
Outreach Services	Fly-in, fly-out Hub-and-spoke
Virtual Outreach Services (telehealth)	Telehealth such as phone review, web-based, consults, remote patient monitoring and virtual clinics.

### Quality assessment and risk of bias in individual studies and across studies

Two researchers independently quality appraised each article using The Mixed Method Appraisal Tool (MMAT). The MMAT is used to appraise the methodological quality of qualitative, quantitative and mixed method reviews [23]. While methodological criteria are more difficult to assess, it may be considered more rigorous than simply reporting quality [24]. Articles were assessed with two screening questions and five quality criteria questions, all articles were then given a rating for each criteria, rather than a total score. Scoring of five quality criteria questions, with a score given as; ‘no’, ‘can’t tell’ or ‘yes’. Studies were considered either high or low quality based on the number of ‘yes’ responses given, with more ‘yes’ responses indicating higher quality. The MMAT also identified risk of bias of individual studies through the criteria questions. A description of the questions used to determine the quality of each study is included in Table 4.

**Table 4:** Summary of mixed methods appraisal tool (MMAT) evaluation criteria [24]

**MMAT Screening Questions** S1 = Are there clear research questions? S2 = Do the collected data address the research questions? **MMAT Criteria for Qualitative Studies** 1.1 = Is the qualitative approach appropriate to answer the research question? 1.2 = Are the qualitative data collection methods adequate to address the research question? 1.3 = Are the findings adequately derived from the data? 1.4 = Is the interpretation of results sufficiently substantiated by data? 1.5 = Is there coherence between qualitative data sources, collection, analysis and interpretation? **MMAT Criteria for Non-Randomised Studies** 3.1 = Are the participants representative of the target population? 3.2 = Are measurements appropriate regarding both the outcome and intervention (or exposure)? 3.3 = Are there complete outcome data? 3.4 = Are the cofounders accounted for in the design and analysis? 3.5 = During the study period, is the intervention administered (or exposure occurred) as intended? **MMAT Criteria for Quantitative Descriptive Studies** 4.1 = Is the sampling strategy relevant to address the research question? 4.2 = Is the sample representative of the target population? 4.3 = Are the measurements appropriate? 4.4 = Is the risk of nonresponse bias low? 4.5 = Is the statistical analysis appropriate to answer the research question? **MMAT Criteria for Mixed Methods Studies** 5.1 = Is there an adequate rationale for using a mixed methods design to address the research question? 5.2 = Are the different components of the study effectively integrated to answer the research question? 5.3 = Are the outputs of the integration of qualitative and quantitative components adequately interpreted? 5.4 = Are divergences and inconsistences between quantitative and qualitative results adequately addressed? 5.5 = Do the different components of the study adhere to the quality criteria of each tradition of the methods involved?	

### Synthesis of results

All studies were reported narratively. All studies were categorised to an existing typology of rural and remote primary health care models [11], where there was a clear fit. The primary aim of the PHC model was summarised. This included measures or descriptions of service availability, service delivery method/s, consistency/regularity of services and services being “fit for purpose”. As per the aims, the impact and effectiveness of PHC models was reported, in addition to any other key outcomes and implications of the models.

## Results

### Study selection

**Fig. 1:**
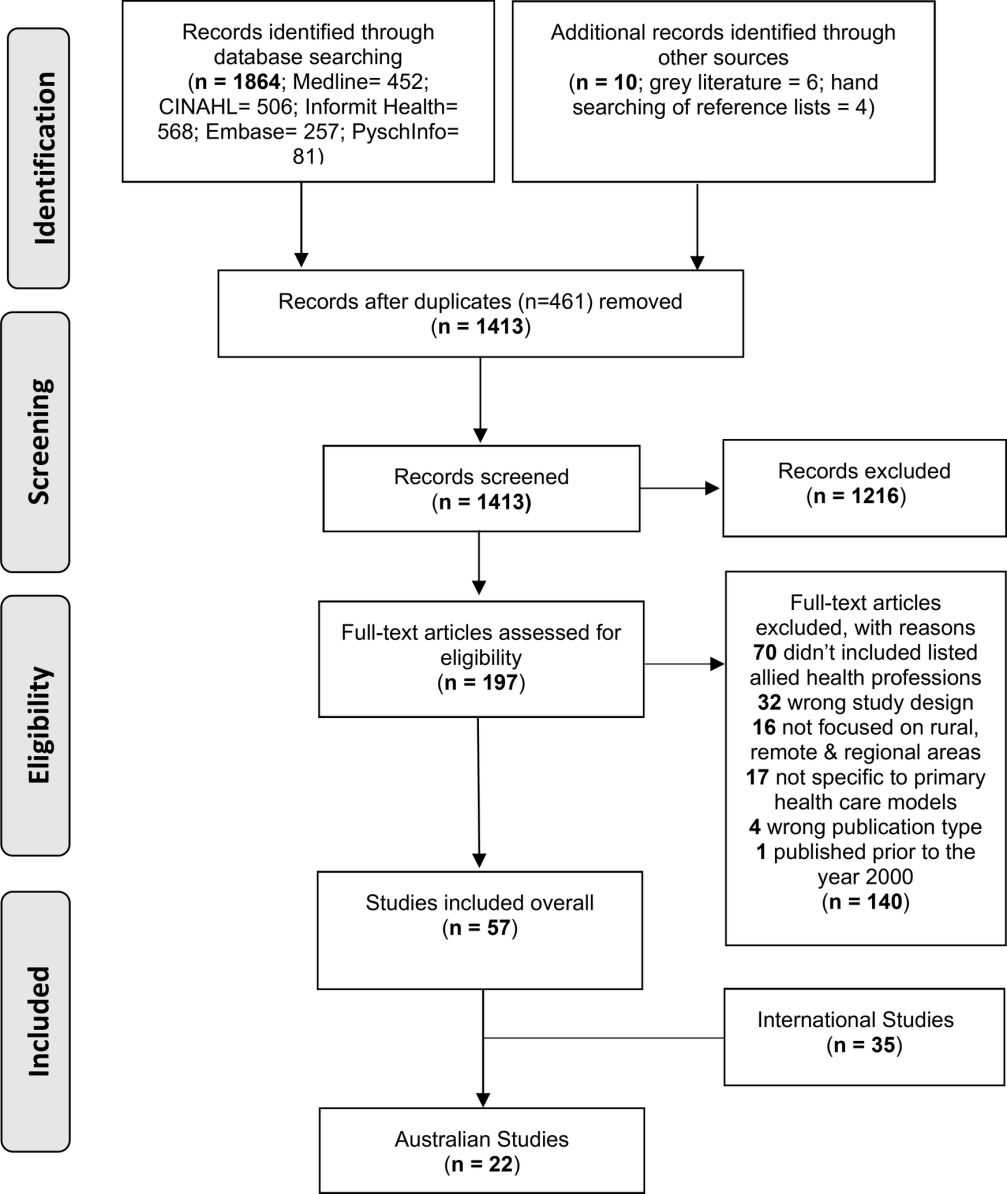
PRISMA flow chart for systematic review of primary health care service delivery models relevant to allied health practice in rural and remote areas of Australia

A total of 1864 potentially eligible articles were retrieved from the five databases. An additional six studies were retrieved from the grey literature and a further four studies identified after hand searching the reference lists of included articles. After 461 duplicates were removed, 1413 article abstracts and titles were screened. A total of 197 articles were included in the full text stage. From the full text screening, 57 articles met the inclusion criteria for the initial review. For this paper, a total of 23 articles (22 studies) have been reviewed, as they relate to the Australian context, as per the aim of this review. The study selection process is summarised in the PRISMA flow chart, shown in Fig. 1.

### Study characteristics

Of the 22 included Australian studies, most were located in four Australian states: New South Wales (*n* = 6) [25–30]; Victoria (*n* = 5) [29, 31–34]; Queensland (*n* = 5) [35–40]; and South Australia (*n* = 4) [41–44]; with one study across two states [29]. Additionally, there was a study conducted in Western Australia [45] and two studies that did not identify the state [46, 47]. Surveys (*n* = 9) were the most common method used to measure service outcomes [28, 29, 32–34, 38, 44, 46, 47]; followed by interviews and/or focus groups (*n* = 6) [26, 30, 36, 40, 42, 43]; and health measurements (*n* = 3) [25, 27, 34]. Studies either focussed on specific health conditions (*n* = 9), such as diabetes [25, 32, 34], cardiovascular health [30], asthma [27], ear health [39] or mental health [29, 41, 47]; or age specific services (*n* = 8), such as paediatrics [26, 28, 34, 35, 38, 40, 42] or older adults [44, 45]; or general health needs for remote communities (*n* = 5) [31, 33, 36, 43, 46]. The majority of studies were able to be mapped to at least one of the existing typology of primary health care service models, as described in Table 3 including integrated services (*n* = 9) [29, 31, 32, 34, 37, 38, 40–43]; outreach services (*n* = 2) [35, 36]; virtual outreach services (*n* = 4) [26, 33, 38, 40, 45]; and discrete services (*n* = 3) [30, 39, 46]. A further model of service delivery, not included in the existing typology, health promotion, was also identified (*n* = 5) [25, 27, 28, 44, 47]. Of the included studies, occupational therapy (*n* = 10) [26, 33, 35–38, 40–42, 44, 46], dietetics (*n* = 8) [25, 26, 33, 34, 36, 37, 41, 43], psychology (*n* = 7) [25, 26, 29, 31, 36, 42, 45], and podiatry (*n* = 7) [30–32, 36, 37, 41, 43] were the top four allied health disciplines involved. A summary of the individual studies is provided in Tables 5 and 6.

**Table 5:** Summary of the PHC models used by allied health professionals in non-metropolitan areas, *n* = 22^

Author/s (year) [ref no.]	Town, State Rurality classification*	Allied Health Professions	Target Group	Type and method of Primary Health Care Service**	Aim of the PHC model
Agostino J., et al. (2012) [35]	Cape York, Queensland *Remote*	Occupational Therapy and other allied health professionals (not specified in the article)	Paediatrics	*Outreach services*: *FIFO* Team based approach to healthcare including clinics, case conferences and health promotion activities.	Access to services - (increase in primary healthcare to manage acute and chronic illness)
Almeida PO., et al. (2021) [45]	Regional and remote regions of Western Australia (as per ASGC-RA Classification) *Regional/Remote*	Psychology	Older adults (65 years or older) with subthreshold depressive symptoms	*Virtual outreach service*: phone	Access to services
Asaid A., et al. (2007) [31]	Elmore, Victoria *MM5*	Psychology, physiotherapy, podiatry	Small rural and remote communities	*Integrated services*: PHC team (multidisciplinary) The foundation model is a community coordination & outreach service model with a combination of public and private health funding. A single-entry point (via GP) to a locally appointed service coordination manager and practice nurse to enable access to services including allied health	Access to service - service availability
Battye KM., et al. (2003) [36]	Remote communities in North-West Queensland *Remote*	Physiotherapy, podiatry, dietetics, occupational therapy, speech pathology, psychology	11 culturally diverse (Indigenous, non-Indigenous and mixed) remote communities	*Outreach services*: hub-and-spoke	Access to services - service availability, frequency and consistency of services
Bergin SM., et al. (2009) [32]	Victoria *Rural/regional*	Podiatry	Diabetes-related foot disorders	*Integrated services*: PHC teams (multidisciplinary) Comprised of medical and allied health professionals providing community based ongoing podiatry care	Access to services
Cairns A., et al. (2024) [37]	Western Cape York, Queensland *MM7*	Physiotherapy, dietetics, speech pathology, social work, occupational therapy, podiatry, allied health students	Remote communities	*Integrated services:* PHC team (multidisciplinary) 3 tiers: 1 - community and the Service working together to build capacity; 2 - co-facilitation of group programs by the Service and community organisations for prevention and intervention; 3 - delivery of individual rehabilitation or specialist care, coordinated through the Service.	Access to services - services being fit for purpose to suit location/population that complement the primary healthcare services already being delivered, and focus on workforce and community capacity building
Ervin K., et al. (2021) [33]	Northern Victoria *Rural*	Allied or community health workers including: dietetics, physiotherapy, health promotion, occupational therapy, social work	Health professionals including allied health/community health	*Virtual outreach service*: telehealth	Service delivery method- access to services that would not otherwise be provided
Fairlamb J., et al. (2007) [41]	Murray Bridge, South Australia *Regional*	Dietetics, podiatry, speech pathology, occupational therapy, physiotherapy, social work	Mental Health	*Integrated services:* PHC teams (multidisciplinary) Health & wellbeing team model works towards practical outcomes in people’s lives.	Access to services- there is a waiting list for services and fear that acute mental health services will not be provided.
Goss PW., et al. (2010) [34]	Sale, Victoria *MM4*	Dietetics	Paediatric diabetes	*Integrated services*: PHC team (multidisciplinary) Multi-disciplinary clinic- grouping patients into three monthly clinics	Access to services- access to an increased variety of allied health services.
Harris C., et al. (2005) [25]	200 km south of Wollongong, NSW *Rural*	Dietetics, psychology	Diabetes	*Health promotion*: Culturally appropriate health promotion 5-day camp to support self-management. Daily workshops focused on disease management, physical activity, nutrition, stress management, relaxation training & smoking cessation.	Access to services
Hawke M., et al. (2000) [42]	Victor Harbour, Southern Fleurieu, South Australia MM3	Occupational therapy, Speech pathology, physiotherapy, psychology	Paediatrics	*Integrated services*: PHC team (multidisciplinary) Transdisciplinary early intervention model with case management approach; run out of a community health centre with some home visits.	Access to services- the provision of early childhood services to a population that previously had no access to services
Lewis P., et al. (2003) [43]	Whyalla, South Australia *MM3* Port Lincoln, South Australia *MM6*	Dietetics, podiatry	Rural and remote communities	*Integrated services*: PHC team (multidisciplinary) Enhanced Primary Care funding to complete care plan to assist people with chronic illness and complex care needs- facility based and home based.	Access to services- increasing management and coordination of care for patients with multidisciplinary team needs.
Luscombe GM., et al.(2021) [26]	Orange (Hub site) and Canowindra, Condobolin, Cowra, Forbes, Grenfell, Molong and Parkes (spoke sites), New South Wales *MM3 - 6*	Speech pathology, dietetics, psychology, occupational therapy, child and family health nurses	Paediatric feeding clinic	*Virtual outreach service*: telehealth Hub and Spoke	Virtual model - supporting continuity of care, a translatable model of care, supporting therapeutic relationships between specialists and families
Merritt et al. (2013) [46]	Regional and Remote Australia Outer regional *(RA3)*, *remote (RA4) or very remote (RA5)*	Occupational therapy	Outer regional, rural and remote Occupational Therapists	*Discrete and outreach services*: Private practice- including outreach services to smaller towns, surveyed regarding location and type of services provided, practice models and demographics.	Access to services - service availability
Misan G., et al. (2018) [44]	Whyalla, South Australia *MM3*	Occupational therapy students	Older men	*Health promotion*: Student-led education sessions Project 1- healthy eating & nutrition. Project 2- physical activity & exercise. Education sessions, skills development sessions, practical sessions, survey on current lifestyle choices.	Access to services Service delivery method- being education sessions
Phillips D., et al. (2021) [38]^	Rural or remote communities (> 30 min drive) outside Townsville, Queensland *Rural/remote*	Specialist occupational therapy	Therapy (OT)-Led Paediatric Burn Telehealth Review Clinic (OTPB Clinic)	*Virtual outreach services: t*elehealth from extended scope OT *Integrated services*: shared care between local services and virtual outreach provider/allied health assistants where available	Access to services
Phillips D., et al. (2022) [40]^	As above	As above	As above	As above	Continuity of care, family-centred care, technology and rural capacity building
Raynor AJ., et al. (2024) [47]	21 rural and remote regions across Australia *Rural/remote*	Exercise physiology	Mental health	*Health promotion:* 8 week program includes one hour of supervised group exercise per week, followed by a one-hour lifestyle education session	Access to services Service delivery method - inclusion of HEALing Mental Health program within education sessions
Saini B., et al. (2008) [27]	Central West NSW Orange and Dubbo *Rural/regional*	Pharmacy	Asthma	*Health promotion/prevention*: RAMS model is an asthma care model with health promotion, screening and disease management services.	Access to services
Skinner J., et al. (2021) [28]	Rural and regional areas of New South Wales *Rural/regional*	Oral health therapy	Paediatrics	*Discrete services*: Oral health service delivery *Health promotion*: Oral health promotion in schools (dental screenings and referrals, education, school physical restructuring, resource distribution)	Access to services - increasing access to dental professionals in rural and remote areas; additional oral health service delivery.
Taylor S., et al. (2021) [39]	Two Rural community pharmacies Queensland *MM4 - 5*	Pharmacy	LISTEN UP program, an innovative model, expanding services offered by rural community pharmacies for ear complaints	*Discrete services*: Community pharmacist provides otoscopy examination, tympanometry, hearing screening and basis assessment and recommends appropriate treatment or referral to GP	Access to services
Vines RF., et al. (2004) [29]	Bathurst, Rylstone, Trundle and Armidale, NSW and Ballarat, Victoria *Regional/rural/remote*	Psychology	Mental Health	*Integrated services*: shared care (collaborative model) The intervention comprised six sessions- full assessment, case formulation and choice of relevant psychological interventions.	Access to services
Warner P., et al. (2010) [30]	Albury, NSW Wodonga, NSW *Rural*	Podiatry students	Adults from the regional community for cardiovascular risk assessment	*Discrete services*: university student- led clinics (specific health unit) University student-led clinic groups of students from two disciplines assigned to a station.	Access to services- no cost to participants Service availability - providing access to a community cardiovascular screening program

*Rurality has been reported as per the authors description in the paper or if specific locations were reported, the Modified Monash Model was used: MM2, regional centres; MM3, large rural towns; MM4, medium rural towns; MM5, small rural towns; MM6, remote communities; MM7, very remote communities. ** links to Typology in Table 3. ^ Phillips et al. [38] and Phillips et al. [40] are from the same study

**Table 6:** Summary of the outcomes of the studies on PHC models used by allied health professionals in non-metropolitan areas, *n* = 22^

Author/s (year) [ref no.]	Methods of study	Study Participants	Impact or Effectiveness outcomes	Other Key Outcomes	Implications
Agostino J., et al. (2012) [35]	Description of Cape York Paediatric Outreach Clinic	N/A	N/A	Sustainability of model- effective way of maintaining staff & increasing services	FIFO currently used and associated with low staff turnover. Authors suggest the ideal is locally based staff through training of community members to be indigenous health workers
Almeida PO., et al. (2021) [45]	RCT with intervention consisting of self-managed behavioural activation program supported by three 45-min phone sessions delivered by a trained psychologist over a period of 8 weeks. Also included self-help booklet.	309 older adults in regional, rural, remote areas of Western Australia who were screened for disordered mood.	Effective at decreasing the severity of depressive and anxiety symptoms over a period of 12 months compared to controls who had no phone support or booklet with behavioural activation strategies.	N/A	Unable to establish the clinical significance of the improvement in depressive and anxiety symptoms. However this type of intervention may have a role in improving mental health outcomes for older people living in regional and remote areas.
Asaid A., et al. (2007) [31]	Description of the evolution of the “Elmore Model of Primary Health Care”	N/A	N/A	N/A	This model ensured all services available were being used, including government or privately funded services. The model combines the benefits of local coordination and integration of general practice and other primary health services, and places the community at the centre of all development, planning, and service delivery processes.
Battye KM., et al. (2003) [36]	Description of the establishment of a model including the steps: Development of a planning matrix; Environmental scan (including mapping/gap analysis; Community consultation (Focus groups and interviews via telehealth or face to face) analysis of morbidity and mortality data); Desktop analysis; Synthesis of information to develop a model	Members of the community 12 allied health professionals	N/A	N/A	Final model suggested was a hub-and-spoke model with allied health services outreaching from the hub (Mt Isa) into each of the three geographically separate areas (spokes). The effectiveness of the model has yet to be evaluated.
Bergin SM., et al. (2009) [32]	State-wide survey- Footcare Provider Survey sent to community health centres	*n* = 69 responses from community health clinics; *n* = 45 from rural and regional areas	N/A	Access to services– 88% provided ongoing podiatry care to individuals with diabetes, 8 (11.6%) indicated no clinical podiatry care was provided of which 7 were in rural or regional areas. 7 community health centers provided no podiatry services at all, of which 6 were rural or regional services.	Identified barriers to providing care included staffing issues, lack of resources or knowledge from health professions on a podiatrists role in managing diabetes related foot conditions.
Cairns A., et al. (2024) [37]	Description of a co-designed Integrated Allied Health Service model	N/A	N/A	N/A	There is a need for a more collective approach between health and social services to facilitate pooling resources in rural and remote communities with limited resources to delivery consistent quality care. Models that include student placements are both a rural workforce recruitment strategy and can address health service gaps in remote communities with a limited local allied health workforce.
Ervin K., et al. (2021) [33]	Electronic survey to understand telehealth practices, purposes and attitudes	*n* = 11 allied health *n* = 13 community health	N/A	53% had reported starting to use telehealth as a result of COVID- 19. 58.3% (*n* = 14) agreed or strongly agreed that they feel telehealth will become a normal part of their work.	Respondents identified there is need for ongoing education and training when using telehealth. Telehealth should not be a replacement for face-to-face services, it should be supplementary.
Fairlamb J., et al. (2007) [41]	Description of current service	N/A	N/A	N/A	Systems and polices need to be developed to support the innovative work that is happening and to increase it in regard to mental health.
Goss PW., et al. (2010) [34]	Health measures & survey	*n* = 56 participants had access to the model of care in 2009, mean age of 14 years	Significant improvement in glycaemic control when compared with 2006 figures.	Satisfaction with service- 89% of participants felt more supported; 86% felt that their diabetes was more controlled with a team approach.	In a rural setting child and adolescent diabetes care can be provided by the multidisciplinary team to achieve positive outcomes, with results comparable to large metropolitan areas.
Harris C., et al. (2005) [25]	Blood pathology changes over time (pre, 3-month, 6 month)	*N* = 20 Aboriginal participants with diabetes	Improved lipid levels Improved Glycaemic control	All strategies were used by more than 50% of the participants. 66% of participants reported they were better able to self-manage their diabetes.	Management of complex and chronic conditions requires a shared care approach to improve outcomes. Camps are an effective way of increasing knowledge of diabetes and self-management strategies in the Aboriginal population.
Hawke M., et al. (2000) [42]	Description and evaluation of the model including service usage and group feedback sessions	Children aged 0–8 years with developmental delay for early intervention	N/A	78 referrals within first 12 months. Sustainability of the model- project was deemed appropriate and efficient and received yearly funding to continue.	The program has emphasised the significance of maintaining a therapeutic relationship with parents in promoting developmental change. The effect of this approach has been to broaden the scope of families receiving services from the Southern Fleurieu Health Service with minimal additional costs.
Lewis P., et al. (2003) [43]	Interviews, questionnaires and observations	*n* = 16 care plans observed across three general practices	N/A	Patients reported better matching of health care services to need, improved quality of care and improved knowledge	Strategies aiming to increase the uptake of enhanced Primary Care items need to address efficiency and accessibility, as well as appropriate remuneration for health professionals.
Luscombe GM., et al.(2021) [26]	Qualitative Inquiry using interviews with clinicians	*N* = 9 health professionals	N/A	Benefits to clinicians: positive benefits of development of professional skills and confidence, experiential learning, improved understanding of roles within an interdisciplinary team and relationship building; Benefits for clients and families: access to a specialist service, convenience/reduced opportunity costs	Hub and spoke virtual model provides increased access to specialist care
Merritt et al. (2013) [46]	23 item survey with open and closed questions of private occupational therapy providers	*n* = 58 occupational therapists based in rural and remote areas	N/A	32 different specialty areas reported, no difference in services provided between outer regional and remote, however no access to neurological rehab, mental health and driving assessments in remote areas. 89% based in outer regional, 11% remote. Very remote towns received visiting services. Most respondents 72% visited 3 towns with one quarter visiting at least 5 towns. Sustainability of model- main sources of income were DVA (68%), workers compensation authorities/insurers (60%), motor vehicle accident insurers (56%), Medicare CDM (56%) and private consultancy (52%). Nearly half the private OT workforce plan to leave private practice in the next 5 years.	Long term sustainability uncertain for OT private practice, potential market failure due to insufficient demand. Hub-and-spoke model is proposed to address this issue.
Misan G., et al. (2018) [44]	Description of an intergenerational learning programs. Including: Literature review; individual survey and questionnaires; group education sessions	Whyalla men’s shed members (*n* = 50 members of which 25 are regular; number of participants not stated)	Improved knowledge on risk factors for chronic disease and strategies to reduce risk; including the importance of maintaining a healthy diet and regular physical activity.	Student benefits: improved knowledge on men’s health and older people and community consultation.	Student led health promotion programs appear to be well suited to this target group in order to increase health knowledge and empower the members.
Phillips D., et al. (2021) [38]^	Evaluation of an occupational therapy led paediatric telehealth burns review clinic Including: using patient satisfaction surveys and number of clinical encounters	*n* = 28 paediatric burns patients attending clinic between Jan– June 2017	Rural children received review every 8 weeks (on average) increased from 20 weeks pre-trial. Travel time of 12 h per family saved. 1 child required surgical review (< 4%).	Satisfaction with service: time saving including no travel, less time off work for parents/school for children, continuity of care from same therapist.	Extended scope role for OT supported quality care while freeing up paediatric surgeons. Follow up appointment frequency improved.
Phillips D., et al. (2022) [40]^	Semi-structured interviews, qualitative approach	Eight family groups Six clinicians	N/A	Four major themes were derived: continuity of care gave families confidence with service, family-centred care, technology and rural capacity building (for clinicians)	This advanced-scope, OT-led telehealth model provided quality patient-centred and expert clinical advice within local communities and builds the skill and capacity of local clinicians.
Raynor AJ., et al. (2024) [47]	Prospective cohort study; measures included 19 individual items organised across seven domains	117 adult participants (99 were included in analysis − 31 males and 68 females); mean age of 59.5 years	The program showed a strong positive effect on participants’ readiness to change, level of physical activity and mental wellbeing	N/A	The provision of a free community-based program was beneficial for those who attended more than 50% of the sessions. The mental health version of the standard HEAL™ program can be used to enhance the engagement and participation of individuals in physical activity.
Saini B., et al. (2008) [27]	Non-randomised controlled trial. Patients visited the pharmacy at baseline, 1, 3 and 6 months after baseline. Questionnaire and asthma severity score was completed.	Intervention pharmacists (*n* = 12) trained to deliver RAMS model, and control pharmacists (*n* = 8) providing standard asthma care from 6 to 8 pharmacies in each site. 51 and 39 patients were recruited by intervention & control pharmacists	Intervention group had significant change in asthma severity score from severe to a moderate.	Intervention pharmacists delivered 362 interventions at the baseline visit (7.7 intervention per patient) and 44 interventions at the final visit (1.0 intervention per patient) spending a mean time of 41.2 +/- 11.5 min per patient at baseline and 15.6 +/− 7.2 min per patient at the final visit. Sustainability of the model- cost savings to the health care system based on a change in severity was estimated to be for the intervention group $5632.70 monthly.	The RAMS model may increase access to services for individuals who have asthma in the primary health care setting. This would improve individual’s ability to self-manage their asthma, as well as allowing for a collaborative approach between the patient and health professional.
Skinner J., et al. (2021) [28]	Online survey with oral health therapists and supervisors in the Dalang Project	15 oral health therapist graduates between 22–28 years of age (13 female, 1 male, 1 other) 4 of the 15 respondents were originally from rural or regional areas.	Improved oral health status and oral hygiene behaviours	A total of 63 schools, 21 preschools and 15 community health services received regular dental health education through the Dalang Project. The Dalang Project was well received by both the oral health professionals and the Aboriginal community controlled health sector (ACCHSs).	The Dalang Project is an example of a successful co-designed project that has positively impacted oral health service delivery for Aboriginal children and has provided a valuable experience for new graduate oral health therapists working in Aboriginal Community Controlled Aboriginal Health Services
Taylor S., et al. (2021) [39]	Pilot mixed method study based on PRECEDE-PROCEED model. Planning, piloting and process evaluating a community pharmacy project for participants with ear complaints.	*n* = 18 adults participants with ear complaints, average age 44 years, two thirds of participants were female.	At seven day follow up, 5 participants symptoms had resolved, 3 were improving and 1 was not improving. 5 participants were referred to GP.	33% couldn’t see GP about ear complaint prior to attending pharmacy, 72% would have attended GP if pharmacy service was not available	Participants recommended service and would go to pharmacy first before seeing a GP for future ear complaints
Vines RF., et al. (2004) [29]	Cohort study: Measures of level of psychological dysfunction assessed before and after the intervention using the DASS, GHQ and GWBI scales.	*n* = 276 general practice patients with mental illness receiving collaborative treatment from GPs and clinical psychologists in comparison with a normative sample of *n* = 198 patients attending the same GP surgeries.	Treatment scores of the intervention group had improved significantly on all DASS, GHQ, and GWBI measures, indicating a positive change in mental health status of the patients.	N/A	Findings suggest that shared care involving GPs and psychologists leads to an improved mental health status in patients.
Warner P., et al. (2010) [30]	Interviews were conducted as well as a questionnaire for students	*n* = 524 adult participants ranging in age from mid- 40’s to mid- 80’s. *n* = 20 students (from nursing and podiatry disciplines)	N/A	Sustainability of model- had been running for four years at the time of publication with participants attending on more than one occasion. Students felt the experience was worthwhile for practicing skills and improving knowledge	Community engagement project has displayed its sustainability and therefore may be used by other Universities as a template to develop a similar program

^ Phillips et al. [38] and Phillips et al. [40] are from the same study. CDM, chronic disease management; DASS, depression, anxiety & stress scale; FIFO, fly-in-fly-out; GP, General practitioner; GHQ, general health questionnaire; GWBI, general well-being index; OT, occupational therapy; RAMS, rural asthma management service

### Quality appraisal & risk of bias

The included studies were assessed across the five categories of the MMAT: randomised studies (*n* = 1) [45]; non-randomised studies (*n* = 7) [25, 27, 29, 34, 42, 46, 47]; quantitative studies (*n* = 4) [28, 30, 32, 33]; qualitative studies (*n* = 4) [26, 40, 43, 44]; and mixed method studies (*n* = 3) [36, 38, 39]. Of the 22 studies, eight studies were found to be of low quality [28, 30, 33, 36, 38, 39, 42, 43] and 11 studies were considered to be high quality [25–27, 29, 32, 34, 40, 44–47]. The studies classified as ‘randomised’ or ‘non-randomised’ were assessed as higher quality compared to the remaining studies. Four studies could not be assessed using the MMAT due to being purely descriptive studies and did not meet the tool’s criteria for evaluation [31, 35, 37, 41]. The MMAT ratings of all 22 studies are listed in Tables 4 and 7.

**Table 7:** Mixed methods appraisal tool results for studies included in this review [24]

Mixed Methods Appraisal Tool Assessment Criteria							
**Author/s** **(year)(ref)**	**Screening** **Questions**		**Criteria for** **Qualitative Studies**				
	S.1	S.2	1.1	1.2	1.3	1.4	1.5
Lewis et al. (2003) [43]	yes	yes	can’t tell	can’t tell	no	no	can’t tell
Luscombe et al. (2021 [26]	yes	yes	yes	yes	yes	yes	yes
Misan et al. (2018) [44]	yes	yes	yes	yes	can’t tell	can’t tell	yes
Phillips D., et al. (2022 [40]	yes	yes	yes	yes	yes	yes	yes
**Author/s** **(year) (ref)**	**Screening Questions**		**Criteria for** **Randomised Controlled Studies**				
	S.1	S.2	2.1	2.2	2.3	2.4	2.5
Almeida et al. (2021) [45]	yes	yes	yes	yes	yes	yes	yes
**Author/s** **(year) (ref)**	**Screening** **Questions**		**MMAT Criteria for** **Non-Randomised Studies**				
	S.1	S.2	3.1	3.2	3.3	3.4	3.5
Goss et al. (2010) [34]	yes	yes	yes	yes	yes	can’t tell	yes
Harris et al. (2005) [25]	yes	yes	yes	yes	yes	can’t tell	yes
Hawke et al. (2000) [42]	yes	yes	yes	can’t tell	can’t tell	no	yes
Merritt et al. (2013) [46]	yes	yes	can’t tell	yes	yes	no	no
Raynor et al. (2024) [47]	yes	yes	can’t tell	yes	yes	can’t tell	yes
Saini et al. (2008) [27]	yes	yes	yes	yes	yes	can’t tell	yes
Vines et al. (2004) [29]	yes	yes	yes	yes	yes	can’t tell	yes
**Author/s** **(year)(ref)**	**MMAT Screening Questions**		**MMAT Criteria for** **Quantitative Descriptive Studies**				
	S.1	S.2	4.1	4.2	4.3	4.4	4.5
Bergin et al. (2009) [32]	yes	yes	yes	yes	yes	can’t tell	yes
Ervin et al. (2021) [33]	yes	yes	yes	can’t tell	yes	can’t tell	can’t tell
Skinner et al. (2021) [28]	yes	yes	can’t tell	yes	yes	can’t tell	N/A
Warner et al. (2010) [30]	can’t tell	can’t tell	no	can’t tell	can’t tell	can’t tell	can’t tell
**Author/s** **(year)(ref)**	**Screening Questions**		**Criteria for** **Mixed Methods Studies**				
	S.1	S.2	5.1	5.2	5.3	5.4	5.5
Battye et al. (2003) [36]	yes	yes	can’t tell	yes	can’t tell	can’t tell	can’t tell
Phillips et al. (2021) [38]	no	can’t tell	yes	can’t tell	no	no	no
Taylor et al. (2021) [39]	yes	yes	yes	yes	no	can’t tell	no

Refer to Table 4 for MMAT Evaluation Criteria and Screening Questions; *n* = 4 studies did not meet the criteria for assessment using MMAT as they report descriptive studies [31, 35, 37, 41]

### Outreach services

Of the 22 studies, three studies were considered outreach services [35, 36, 46]. In terms of rurality, all the studies were considered remote. None of the studies provided clear measures detailing the effectiveness or impact of the primary health care model. Two of the studies, instead, described the PHC model [35, 36]. All of the studies included a range of allied health professions with a primary aim of the PHC model to improve access to services, with two studies also reporting on other key outcomes such as the sustainability of the model [35, 36]. All of the studies used a different type of outreach service, with one study utilising a fly in-fly out model of care whereby allied health professionals and clinicians provide paediatric healthcare to remote communities in Cape York, Queensland [35]. Another study utilised a hub-and-spoke model of care to provide allied healthcare to remote communities in North-West Queensland [36].

### Virtual outreach services

Virtual outreach services were described by four studies (in five publications) [26, 33, 38, 40, 45]. Telehealth was used by all studies as the primary virtual outreach service to assist with the care for older adults with depressive symptoms [45], paediatric feeding clinics [26], paediatric burn review clinic [38, 40] and general access to allied health care [33]. Access to services was the primary aim of the PHC in three studies [33, 38, 40, 45]. Two studies did not report on the impact or effect of the PHC model [26, 33]. Whilst a further two studies reported telehealth support improved depression and anxiety symptoms for older adults in one study [45] and access to a telehealth paediatric burn review clinic improved from reviews every 20 weeks to an average review of every 8 weeks [38, 40] with fewer patients requiring surgical review and higher satisfaction with the telehealth service and continuity of care [38, 40].

### Discrete services

Discrete services were described in four studies [28, 30, 39, 46]. The primary aim of each of these studies were related to access to services with one pharmacy based study increasing access to care for those with ear complaints without having to see a general practitioner [39] and another study reporting an increase in access to services by engaging podiatry students in cardiovascular screening clinics, resulting in a sustainable method of care [30]. A further discrete service study conducted surveys with occupational therapists and reported that 89% of respondents provided care in outer regional areas and the majority regularly visited three regional/rural towns, however other key outcomes of sustainability were uncertain due to insufficient long term demand [46].

### Integrated services

Integrated services were reported in nine studies [29, 31, 32, 34, 37, 38, 40–43]. Multidisciplinary integrated services in a PHC team were used in seven studies [31, 32, 34, 37, 41–43], whilst 2 studies utilised a shared cared PHC model [29, 38, 40]. Access to services was the primary aim for all studies. A study in very remote QLD described its multidisciplinary team of allied health professionals to improve access to care in the region, highlighting the need for a collective fit-for-purpose approach and focus on improving workforce and community capacity building [37]. Similarly, a study in regional South Australia described its multidisciplinary health and wellbeing team to provide mental health services in the region, however, it highlighted the need for further evaluation measures and resources to be provided to appropriately evaluate the impact of the PHC model [41]. Another mental health study conducted in Victoria and New South Wales reported that the integrated shared care PHC model between general practitioners and psychologists, not only improved access to mental health services but significantly improved the mental health status of the patients in the cohort study [29]. Three studies used integrated services in its paediatric care [34, 38, 40, 42]. One study, described in two articles, utilised both integrated services and virtual outreach services in its paediatric burn telehealth review clinic [38, 40]. A multidisciplinary clinic was described in a study for children with diabetes and reported an increase in the variety of allied health services available resulting in higher levels of satisfaction with the care provided [34]. Another paediatric study in regional South Australia also reported providing services via a multidisciplinary PHC team for early intervention using a range of allied health professionals, that the population previously had no access [42].

### Health promotion services

Health promotion services were reported in five of the 22 studies [25, 27, 28, 44, 47]. Access to services was the primary aim of the PHC for four of the five studies [25, 27, 28, 44] with service delivery being the primary aim of one study [47]. All studies reported on the impact of health promotion service. A study of Aboriginal participants with diabetes who received 5 daily workshops to support self-management resulted in improved glycaemic control and lipid levels [25]. Occupational therapy student led education sessions in nutrition and physical activity in older men resulted in improved knowledge of participants [44]. A prospective cohort study of an 8 week health promotion program resulted in a positive effect on the level of physical activity and mental wellbeing [47]. A non-randomised trial for an asthma care model delivered by pharmacists resulted in a significant improvement in asthma severity and access to care [27]. An oral health promotion program in schools increased access to dental professionals and improved oral health status and oral hygiene behaviours [28].

### Other initiatives

Other initiatives that support PHC service delivery were reported in four studies [30, 37, 38, 40, 44]. Three studies focussed on student involvement [30, 37, 44], with two studies specifically describing the use of student-led services in the provision of PHC services [30, 44]. Extended scope of practice was also reported in one study via two articles [38, 40].

## Discussion

This review is the first systematic review to explore allied health primary health care models in rural and remote Australia. There is a range of PHC models for service delivery that have been identified as being used by allied health professionals in rural and remote areas. This review identified that most of the published papers on Australian allied health PHC models fitted within an existing typology of five PHC models [11], with the addition of a health promotion model. Health promotion has not been described in previous typologies due to a predominant emphasis on healthcare treatment, however it is an important consideration in the context of PHC due to its positive impact on chronic disease prevention and behaviour change [48]. These different models provide some guidance to the options available in providing PHC in rural and remote areas. Understanding what PHC models can be used in rural and remote areas remains an important consideration as the prevalence of chronic disease remains higher in these areas compared to metropolitan regions [4]. As such, this review provides important insight into what allied health PHC models are currently being utilised in rural and remote areas.

The evidence of the effectiveness and impact of these models is limited due to limited measures of impact or effectiveness. Many of the articles descriptively reported on the PHC model of care only and did not have clear outcomes and no comprehensive evaluation, indicating the difficulty in exploring the effectiveness and impact of the models of service used. As a result, this review could adequately describe the types of PHC models used by allied health professionals in rural and remote areas of Australia, however, could not determine the impact or effectiveness of most of the PHC models. The implications of this finding suggest more rigorous evaluations of current allied health PHC service models are needed to enable the effectiveness of the models to be explored. There are however challenges to conducting rigorous evaluation of PHC service models that needs to be considered, including funding constraints, methodological challenges, service delivery focus and infrastructure challenges [49]. It also needs to be considered that studies without an evaluative component or research based funding and support are often excluded from peer-reviewed publications, delaying the process of knowledge sharing and translation.

Despite the potential benefits of an extended scope of practice in rural areas, only one study mentioned its use by allied health professionals [38, 40]. This is an interesting finding given that extending the scope of practice of allied health professionals can assist when there is a lack of services. Other researchers have suggested by implementing an extended scope of practices for allied health can lead to an increase to the quality and breadth of health services provided [14]. Research into the use of extended scope of practice in rural nursing has indicated that many legislative and regulatory barriers exist [50].

The development of student-led clinics with oversight by qualified health professionals or by long-arm supervision, has also been promoted to improve access to allied health services in rural areas and has shown positive impact in improve the health and wellbeing of people living in regional, rural and remote areas [51]. Interestingly, few studies reported on this initiative as part of a PHC model despite the potential benefits. Students may provide a valuable contribution to rural based primary care through targeted programs that facilitate their learning and contribute to improvements in primary care services [52].

When considering the limitations of this review, other initiatives such as student led clinics may not have been fully captured, however given the primary focus of the review was on PHC health services and models of care, this limitation needs to be considered within the aims of the study. This review focussed specifically on key allied health professionals only, which may have limited the findings had a broader definition of allied health professionals been included. Similarly, findings for this study included only studies within the Australian context, which may have limited study findings, however it was considered important to highlight Australian-specific research for relevance to the local context.

The present review demonstrates the gaps in the current research regarding allied health PHC models and the effectiveness of the outcomes, with more rigorous evaluations needed. In addition, there is a need to consider policy drivers to support sustained development of allied health services within PHC models. Further, there is an opportunity for future research to explore service delivery models used in an international context by allied health professionals, and for a comparison to those used in the Australian context. However, the review has highlighted suitable models of service delivery and provided clear descriptions that may be utilised in rural and remote settings.

## Supplementary Information

Supplementary Material 1.

## Data Availability

No datasets were generated or analysed during the current study.

## References

[1] World Health Organization. Primary Health Care. 2023. Available from: https://www.who.int/news-room/fact-sheets/detail/primary-health-care.

[2] Australian Institute of Health and Welfare. Primary health care in Australia. Canberra: AIHW; 2016.

[3] Hanson K, Brikci N, Erlangga D, Alebachew A, De Allegri M, Balabanova D, et al. The lancet global health commission on financing primary health care: putting people at the centre. Lancet Global Health. 2022;10(5):e715–72. 35390342 10.1016/S2214-109X(22)00005-5 PMC9005653

[4] Australian Institute of Health and Welfare. Rural and remote health. Canberra: AIHW; 2024.

[5] Gizaw Z, Astale T, Kassie GM. What improves access to primary healthcare services in rural communities? A systematic review. BMC Prim Care. 2022;23(1):313. 36474184 10.1186/s12875-022-01919-0 PMC9724256

[6] NSW Agency for Clinical Innovation. Understanding the process to develop a Model of Care: an ACI framework. 2013.

[7] Carey TA, Wakerman J, Humphreys JS, Buykx P, Lindeman M. What primary health care services should residents of rural and remote Australia be able to access? A systematic review of core primary health care services. BMC Health Serv Res. 2013;13(1):178. 23683166 10.1186/1472-6963-13-178 PMC3663724

[8] Murphy P, Burge F, Wong ST. Measurement and rural primary health care: a scoping review. Rural Remote Health. 2019;19(3):4911. 31365828 10.22605/RRH4911

[9] Thomas SL, Wakerman J, Humphreys JS. Ensuring equity of access to primary health care in rural and remote Australia - what core services should be locally available? Int J Equity Health. 2015;14:111. 26510998 10.1186/s12939-015-0228-1 PMC4625941

[10] Australian Government. Department of Health and Aged care. National Strategic Framework for Rural and Remote Health. Canberra: ACT; 2020.

[11] Wakerman J, Humphreys JS, Wells R, Kuipers P, Entwistle P, Jones J. Primary health care delivery models in rural and remote Australia– a systematic review. BMC Health Serv Res. 2008;8(1):276. 19114003 10.1186/1472-6963-8-276 PMC2642801

[12] Services for Australian Rural and Remote Allied Health. Models of allied health care in rural and remote health. Position Paper. 2016. Available from: https://sarrah.org.au/sites/default/files/docs/models_of_care_may_2016_final.pdf#:~:text=SARRAH%20has%20broadly%20identified%2010%20models%20of%20allied,Local%20community%20multi%20-disciplinary%20primary%20health%20team%3B%205.

[13] Queensland Government. Clinical Excellence Division. Brisbane: Allied Health Expanded Scope Strategy; 2016.

[14] O’Sullivan BG, Worley P. Setting priorities for rural allied health in Australia: a scoping review. Rural Remote Health. 2020;20(2):5719. 32563237 10.22605/RRH5719

[15] National Rural Health Commissioner. Review of rural allied health evidence to inform policy development for addressing access, distribution and quality. Canberra: ACT; 2020.

[16] National Rural Health Commissioner. Improvement of Access, Quality and Distribution of Allied Health Services in Regional, Rural and Remote Australia. Canberra: ACT; 2020.

[17] Bradford NK, Caffery LJ, Smith AC. Telehealth services in rural and remote Australia: a systematic review of models of care and factors influencing success and sustainability. Rural Remote Health. 2016;16(4):3808. 27744708

[18] Speyer R, Denman D, Wilkes-Gillan S, Chen YW, Bogaardt H, Kim JH, et al. Effects of telehealth by allied health professionals and nurses in rural and remote areas: A systematic review and meta-analysis. J Rehabil Med. 2018;50(3):225–35. 29257195 10.2340/16501977-2297

[19] Liberati A, Altman DG, Tetzlaff J, Mulrow C, Gøtzsche PC, Ioannidis JP, et al. The PRISMA statement for reporting systematic reviews and meta-analyses of studies that evaluate healthcare interventions: explanation and elaboration. BMJ. 2009;339:b2700. 19622552 10.1136/bmj.b2700 PMC2714672

[20] Whitford D, Smith T, Newbury J. The South Australian allied health workforce survey: helping to fill the evidence gap in primary health workforce planning. Aust J Prim Health. 2012;18(3):234–41. 23069367 10.1071/PY11027

[21] Australian Government Department of Health. Modified Monash Model. 2020. Available from: https://www.health.gov.au/sites/default/files/documents/2020/07/modified-monash-model-fact-sheet.pdf.

[22] Kellermeyer L, Harnke B, Knight S. Covidence and Rayyan. J Med Libr Assoc. 2018;106(4):580–3. 10.5195/jmla.2018.513. Epub Oct 1.

[23] Pluye P, Gagnon MP, Griffiths F, Johnson-Lafleur J. A scoring system for appraising mixed methods research, and concomitantly appraising qualitative, quantitative and mixed methods primary studies in mixed studies reviews. Int J Nurs Stud. 2009;46(4):529–46. 19233357 10.1016/j.ijnurstu.2009.01.009

[24] Hong QN, Fàbregues S, Bartlett G, Boardman F, Cargo M, Dagenais P, et al. The mixed methods appraisal tool (MMAT) version 2018 for information professionals and researchers. Educ Inform. 2018;34(4):285–91.

[25] Harris C, Curtis O. Supporting Self-management of diabetes in aboriginal people living with diabetes through a 5 day residential camp. Aboriginal Islander Health Worker J. 2005;29(3):4–11.

[26] Luscombe GM, Hawthorn J, Wu A, Green B, Munro A. Empowering clinicians in smaller sites’: A qualitative study of clinician’s experiences with a rural virtual paediatric feeding clinic. Aust J Rural Health. 2021;29(5):742–52. 34490941 10.1111/ajr.12781

[27] Saini B, Filipovska J, Bosnic-Anticevich S, Taylor S, Krass I, Armour C. An evaluation of a community pharmacy-based rural asthma management service. Aust J Rural Health. 2008;16(2):100–8. 18318852 10.1111/j.1440-1584.2008.00975.x

[28] Skinner J, Dimitropoulos Y, Moir R, Johnson G, McCowen D, Rambaldini B, et al. A graduate oral health therapist program to support dental service delivery and oral health promotion in aboriginal communities in new South Wales, Australia. Rural Remote Health. 2021;21(1):5789. 33497576 10.22605/RRH5789

[29] Vines RF, Thomson D, Kluin M, Vesely L, Richards JC, Brechman-Toussaint M. Clinical psychology in general practice: a cohort study. Med J Aust. 2004;181(2):74–7. 15257641 10.5694/j.1326-5377.2004.tb06177.x

[30] Warner P, Jelinek HF, Davidson PM. A university clinic: an innovative model for improving clinical practice. Australian J Adv Nurs. 2010;27(4):38–42.

[31] Asaid A, Riley K. From solo practice to partnering - the evolution of the Elmore model of primary health. Aust Fam Physician. 2007;36(3):167–9. 17339982

[32] Bergin SM, Brand CA, Colman PG, Campbell DA. An evaluation of community-based resources for management of diabetes-related foot disorders in an Australian population. Aust Health Rev. 2009;33(4):671–8. 20166917 10.1071/ah090671

[33] Ervin K, Weller-Newton J, Phillips J. Primary healthcare clinicians’ positive perceptions of the implementation of telehealth during the COVID-19 pandemic using normalisation process theory. Aust J Prim Health. 2021;27:158–62. 33653506 10.1071/PY20182

[34] Goss PW, Paterson MA, Renalson J. A ‘radical’ new rural model for pediatric diabetes care. Pediatr Diabetes. 2010;11(5):296–304. 19895408 10.1111/j.1399-5448.2009.00594.x

[35] Agostino J, Heazlewood R, Ruben A. Cape York paediatric outreach Clinic - improving access to primary care in the cape York Peninsula region. Aust Fam Physician. 2012;41(8):623–5. 23145408

[36] Battye KM, McTaggart K. Development of a model for sustainable delivery of outreach allied health services to remote north-west Queensland, Australia. Rural Remote Health. 2003;3(3):194. 15882095

[37] Cairns A, Rodda D, Wymarra F, Bird K. Healthy ageing in remote Cape York: a co-designed integrated allied health service model. Aust J Prim Health. 2024;30:PY23135. 10.1071/PY23135. 10.1071/PY23135 38237265

[38] Phillips D, Matheson L, Pain T, Kingston GA. Development of an occupational-therapy-led paediatric burn telehealth review clinic. Rural Remote Health. 2021;21(3):6223. 34392690 10.22605/RRH6223

[39] Taylor S, Cairns A, Glass B. Developing an ear health intervention for rural community pharmacy: application of the PRECEDE-PROCEED model. Int J Environ Res Public Health. 2021;18(12):6456. 34203663 10.3390/ijerph18126456 PMC8296273

[40] Phillips D, Matheson L, Pain T, Kingston GA. Evaluation of an occupational therapy led paediatric burns telehealth review clinic: exploring the experience of family/carers and clinicians. Rural Remote Health. 2022;22(1):6887. 35138867 10.22605/RRH6887

[41] Fairlamb J, Muir-Cochrane E. A team approach to providing mental health services in a regional centre using a comprehensive primary health care framework. Aus E J Adv Ment Health. 2007;6(1):5–14.

[42] Hawke M, Byrne J. Community-based early childhood assessment and intervention in rural settings: transdisciplinary case management of developmental delay in children. Aust J Prim Health. 2000;6:130–40.

[43] Lewis P, Misan G, Harvey P, Connolly J, White A, Noone J. Enhanced primary care. A rural perspective. Aus Fam Physician. 2003;32(3):186. 12666361

[44] Misan G, Ellis BJ, Hutchings OM, Beech AK, Moyle C, Thiele NJ. Teaching Old Dogs New Tricks: Health Promotion Through Intergenerational Learning in a Regional Men’s Shed. Aus Int J Rural Educ. 2017;28(1):3–16.

[45] Almeida OP, Patel H, Kelly R, Ford A, Flicker L, Robinson S, et al. Preventing depression among older people living in rural areas: A randomised controlled trial of behavioural activation in collaborative care. Int J Geriatr Psychiatry. 2021;36(4):530–9. 33098159 10.1002/gps.5449

[46] Merritt J, Perkins D, Boreland F. Regional and remote occupational therapy: a preliminary exploration of private occupational therapy practice. Aust Occup Ther J. 2013;60(4):276–87. 23888978 10.1111/1440-1630.12042

[47] Raynor AJ, Nimphius S, Kadlec D, Casson S, Fox-Harding C, Fortington LV. Evaluation of the HEAL™ing Mental Health program: A prospective cohort study of short-term changes from a physical activity and lifestyle education program for people with mental health disorders living in rural Australia. PLoS One. 2024;19(3):e0299859 http://europepmc.org/abstract/MED/38478517 https://journals.plos.org/plosone/article/file?id=10.1371/journal.pone.0299859&type=printable 10.1371/journal.pone.0299859 https://europepmc.org/articles/PMC10936783 https://europepmc.org/articles/PMC10936783?pdf=render. 38478517 10.1371/journal.pone.0299859 PMC10936783

[48] Bisak A, Stafström M. Unleashing the potential of health promotion in primary care-a scoping literature review. Health Promot Int. 2024;39(3):1–10. 10.1093/heapro/daae044. 10.1093/heapro/daae044 PMC11127486 38795052

[49] Bonfim D, Belotti L, de Almeida LY, Eshriqui I, Velasco SRM, Monteiro CN, et al. Challenges and strategies for conducting research in primary health care practice: an integrative review. BMC Health Serv Res. 2023;23(1):1380. 38066627 10.1186/s12913-023-10382-1 PMC10709868

[50] Smith T, McNeil K, Mitchell R, Boyle B, Ries N. A study of macro-, meso- and micro-barriers and enablers affecting extended scopes of practice: the case of rural nurse practitioners in Australia. BMC Nurs. 2019;18:14. 30976197 10.1186/s12912-019-0337-z PMC6444450

[51] Maple M, O’Neill K, Gartshore S, Clark J, White J, Pearce T. School-based multidisciplinary student-led clinics in health and Australian accreditation standards: A scoping review. Aust J Rural Health. 2023;31(6):1168–83. 37888895 10.1111/ajr.13051

[52] Walker C, Forbes R, Osborn D, Lewis PA, Cottrell N, Peek S, et al. The transformation of a student-led health clinic in rural Australia from a face-to-face service to a telehealth model: evaluation of student and client experiences during a covid-19 driven transition. Focus Health Prof Educ Multi Prof J. 2022;23(2):79–92.

